# Severe coagulopathy caused by cefminox sodium in a liver cirrhosis patient: a case report

**DOI:** 10.1186/s13027-022-00446-y

**Published:** 2022-06-16

**Authors:** Shuling Wu, Xiaoyue Bi, Yanjie Lin, Liu Yang, Minghui Li, Yao Xie

**Affiliations:** 1 Department of Hepatology Division 2, Beijing Ditan Hospital, Capital Medical University, No. 8 Jingshun East Street, Chaoyang District Beijing, 100015 China; 2 Department of Hepatology Division 2, Peking University Ditan Teaching Hospital, No. 8 Jingshun East Street, Chaoyang District Beijing, 100015 China

**Keywords:** Cefminox sodium, Coagulation dysfunction, Peritonitis

## Abstract

Cefminox sodium is an antimicrobial agent with broad-spectrum antibacterial activity against Gram-positive and Gram-negative bacteria. Cefminox sodium has high security in clinical practice for its few adverse effects such as coagulation dysfunction, which is rare in clinical treatment. Even in patients suffering from chronic liver disease with coagulation dysfunction, it rarely leads to further deterioration of coagulation function. Therefore, patients with chronic liver disease develop severe coagulation dysfunction during the application of cefminox sodium, which is often mistaken for worsening of liver disease other than considered to be the side effect of the drug. Therefore, we report a 55-year-old female patient with liver cirrhosis and hepatocellular carcinoma treated with cefminox sodium intravenously twice for peritonitis. During the treatments, severe coagulopathy occurred, and the coagulation function quickly recovered after drug withdrawal. The diagnosis and treatment of this patient provides us with ideas for dealing with similar problems in clinical practice in the future.

## Background

The antimicrobial spectrum and antibacterial activity of cefminox sodium are similar to those of the second generation cephalosporins, but the antibacterial activity of cefminox sodium to anaerobic bacteria such as Bacteroides fragilis is stronger than that of the second generation cephalosporins, and it is stable to extended spectrum β lactamases (ESBL), with less probability to develop drug resistance. A therapeutic concentration can be achieved by cefminox sodium in the ascites of patients with peritonitis, so it is widely used in patients with peritonitis [1–3]. Cefminox sodium has high security and few adverse effects, and coagulation dysfunction caused by the drug is rare [4]. Even in chronic liver disease patients with coagulation dysfunction, it rarely leads to further deterioration of coagulation function. Therefore, patients with chronic liver disease develop severe coagulation dysfunction during the period of cefminox sodium treatment, which is often mistaken for worsening of liver disease, rather than the possibility of side effects of the drug, so timely drug withdrawal and rescue measures are seldom considered. The patient with liver cirrhosis reported in our paper was treated with cefminox sodium twice for peritonitis. During each medication, the aggravation of coagulation dysfunction was mistaken for the aggravation of liver disease itself and wasn’t treated in time. Fortunately, it was not life-threatening due to the short time of medication. However, this provides us with a warning role in the clinical diagnosis and treatment of similar cases.

## Case presentation

A 55-year-old woman presented with abnormal liver function on physical examination in 2001 and cirrhosis with small amount of ascites on sonography in 2007. In December 2011, she did abdominal enhanced CT scan in other hospital, and the result showed multiple enhancement foci in her liver. Hepatic arteriography and diagnostic lipiodol embolization were performed in our hospital, and subsequent abdominal plain CT scan showed lipiodol deposition foci in her liver. She was diagnosed as primary liver cancer, and then radiofrequency ablation of liver cancer was performed. Since then, no recurrence of her liver cancer was found during follow-up examinations. However, intermittent fatigue, dizziness, liver discomfort, and edema of the lower extremities began to occur on her since 2013. Due to splenomegaly, hypersplenism, mild hypoproteinemia, a small amount of ascites, esophageal and gastric varices, hepatic encephalopathy and other complications of liver cirrhosis, the patient was repeatedly hospitalized in our department. On May 1, 2018, when she was hospitalized again, her liver and kidney function deteriorated, anemia aggravated, ascites increased significantly, and peritonitis and hepatic encephalopathy occurred intermittently. In addition, the patient had suffered from type 2 diabetes mellitus, diabetic retinopathy, diabetic peripheral neuropathy, chronic renal insufficiency, arrhythmia, paroxysmal atrial fibrillation, osteoporosis, hypocalcemia, hypertrophic osteoarthropathy, anemia, hyperuricemia and other chronic diseases, with long-term use of insulin, Jinshuibao capsule, metoprolol, calcitriol and other drugs, intermittent use of analgesic drugs.

**Fig. 1:**
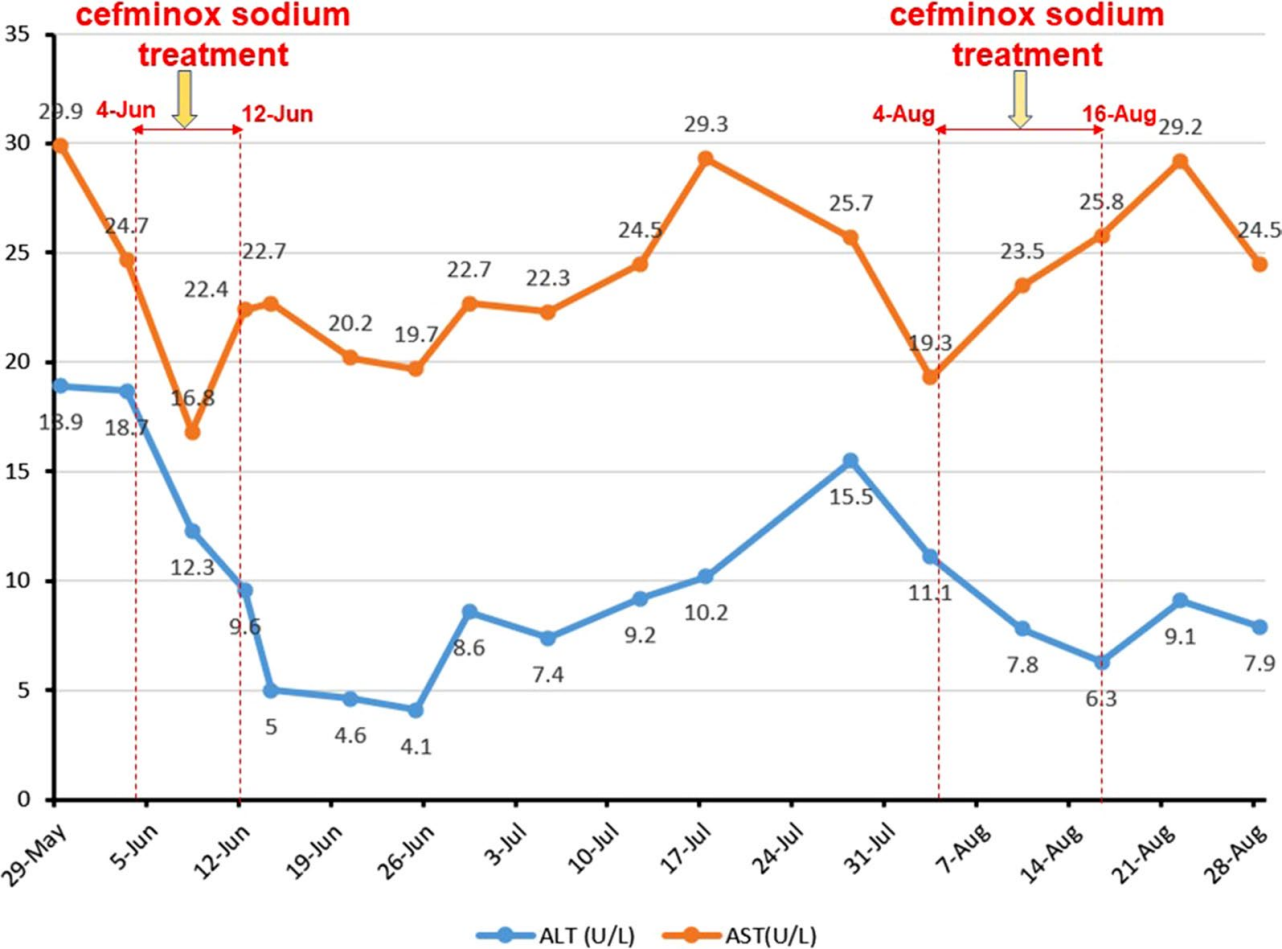
Changes of ALT and AST levels during hospitalization. The ALT and AST levels of the patient were basically in the normal range and occasionally lower than the lower limit of normal during hospitalization

**Fig. 2:**
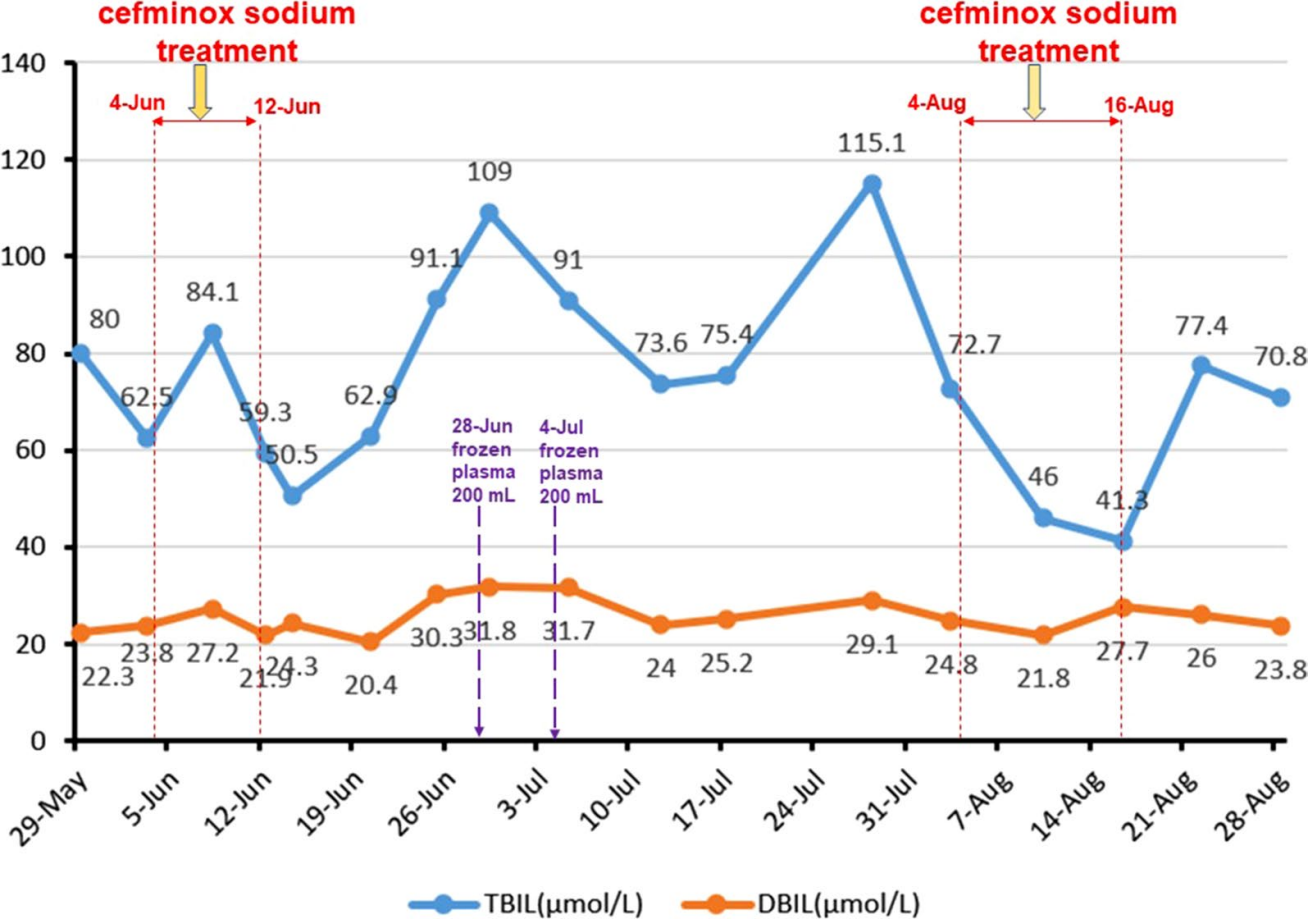
Changes of TBIL and DBIL levels during hospitalization. The patient had obvious hyperbilirubinemia, but the TBIL and DBIL levels didn’t increase during cefminox sodium treatment and didn’t decrease during plasma transfusion. *TBIL* total bilirubin, *DBIL* direct bilirubin

**Fig. 3:**
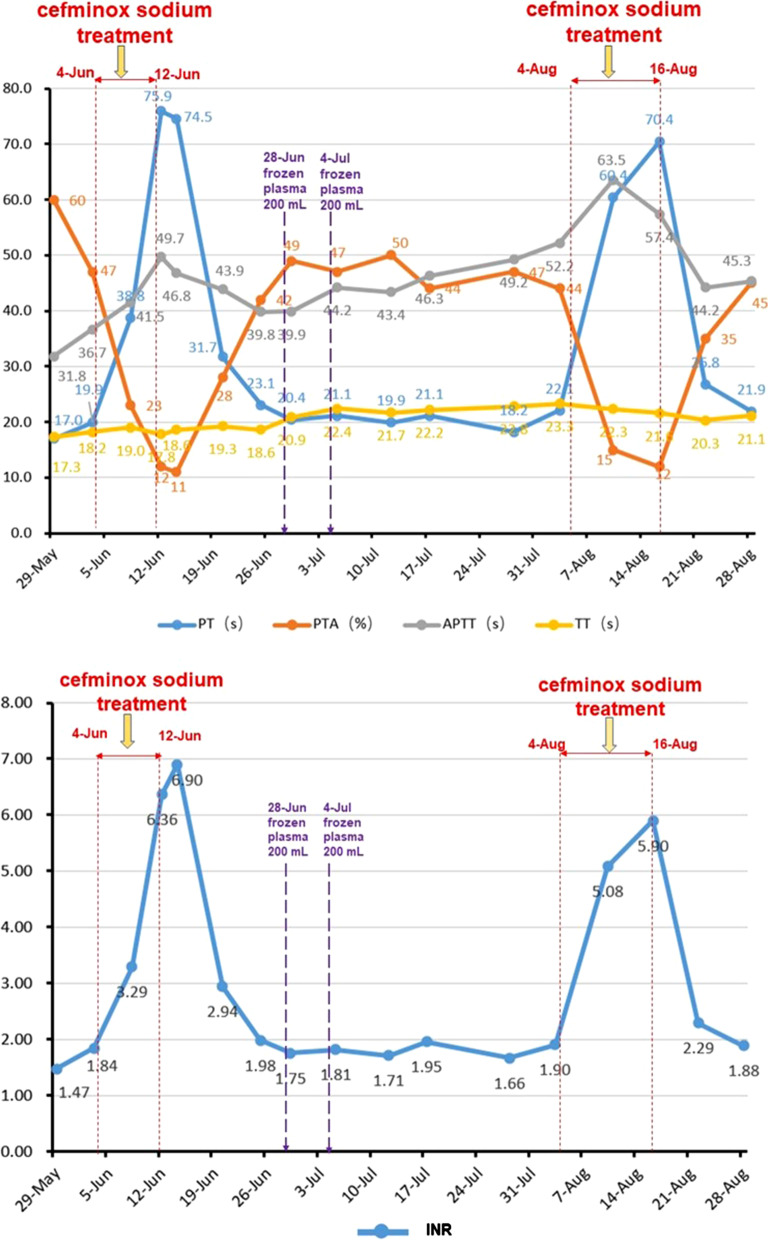
Changes in coagulation function during hospitalization. During cefminox sodium treatment, the patient’s coagulation function deteriorated, which was characterized by prolonged PT and APTT, decreased PTA and increased INR. TT didn’t change significantly. After stopping cefminox sodium treatment, all above parameters quickly returned to the basic levels. *PT* prothrombin time, *APTT* activated partial thromboplastin time, *PTA* prothrombin activity, *INR* international normalized ratio, *TT* bleeding time

Because of peritonitis during hospitalization, the patient was empirically treated with cefminox sodium for anti-infection. The dosage and method were as follows: dissolved 2.0 g of cefminox sodium into 100 mL of 0.9% sodium chloride solution and injected it intravenously every 12 h. Two courses of anti-infection treatment were used. The first use date was from June 4, 2018 to June 12, 2018; and the second use date was from August 4, 2018 to August 16, 2018. During two hospitalizations (including the use periods of cefminox sodium), the alanine aminotransferase (ALT) and aspartate aminotransferase (AST) levels of the patient were basically in the normal range and occasionally lower than the lower limit of normal (Fig. 1). However, the patient had obvious hyperbilirubinemia (Fig. 2), hypoproteinemia and prolonged prothrombin time, mild hepatic encephalopathy and ascites, so her liver function was classified as Child Pugh C at cefminox sodium admission. The parameters of patient’s coagulation function before and after cefminox sodium anti-infection treatment are shown in Fig. 3.

As can be seen from Fig. 3, after the patient started to use cefminox sodium for the first time on June 4, the blood coagulation function tested on June 8 was obviously worse than before: PT and APTT were significantly prolonged, PTA was significantly decreased, and INR was significantly increased. PT was prolonged from 19.9 s before the cefminox sodium usage to 75.9 s on June 12 when cefminox sodium was stopped, which was 2.81 times longer. PTA dropped from 47 to 12%, with a drop of 74.5%. TT didn’t change significantly. Coagulation function didn’t improve after cefminox sodium was discontinued for 2 days, but gradually returned to the original level. The second administration of cefminox sodium was started on August 4, and the same as the first time, the coagulation function deteriorated significantly again. When the cefminox sodium was stopped on August 16, PT was prolonged to 70.4 s again and PTA was decreased to 12%, but the coagulation function of the patient returned to the original level again after stopping the drug.

## Discussion and conclusion

Yasunaga et al. [5] reported the side effect of cefminox sodium on coagulation function for the first time in 1990. PT and APTT were prolonged in 0.29% of patients, fibrinogen (FIB) was decreased in 0.15% of patients, and no thrombocytopenia was found. Patients with underlying chronic diseases, especially those with malignant tumors, were more likely to have coagulation dysfunction after using the drug. In general, coagulopathy caused by cefminox sodium is rare, and few clinical cases have been reported.

As a kind of β-lactam antibiotics, the main cause of coagulation dysfunction caused by cefminox sodium is vitamin K (VitK)-dependent hypoprothrombinemia [6, 7]. Human VitK comes from food intake on the one hand, and from intestinal bacteria synthesis on the other hand. β-lactam antibiotics can inhibit and kill normal intestinal flora, resulting in the reduction of VitK synthesis and the disorder of VitK-dependent coagulation factors (II, VII, IX and X), which results in coagulopathy. Coagulation dysfunction caused by cefoperazone/sulbactam sodium is the most common [8, 9]. In a large retrospective study of 23,242 patients involved, prolonged PT and coagulopathy with cefoperazone/sulbactam were found in 5.3% and 9.2%, respectively, with thrombocytopenia in 15.7% and bleeding in 4.2% of patients [10]. Severe gastrointestinal bleeding can occur in a very small number of patients after the use of cefoperazone [11]. The second reason of coagulation dysfunction caused by cefminox sodium is the *N*-methylthiotetrazole (NMTT) group on the 3rd side chain of cefminox sodium, which directly inhibits mitochondrial shuttling activity or VitK oxidoreductase and affects the VitK-dependent coagulation factor carboxylation, causing VitK-dependent coagulation factor deficiency [12].

No bacterial infection occurred in our reported patient during the previous multiple hospitalizations, and no cefminox sodium was used before. In June 2018, the first time bacterial infection occurred during her hospitalization. According to the patient's abdominal discomfort symptoms, abdominal suspicious tenderness, elevated blood bacterial infection index (total white blood cells, neutrophil ratio, C-reactive protein, procalcitonin), ascites showed by ultrasound, peritonitis was considered. Because the patient refused to perform abdominal paracentesis to extract ascites for laboratory test, she was empirically treated with cefminox sodium. After treatment, the patient's fatigue increased progressively, spirit and appetite were very poor, and the deterioration of coagulation function was mistaken for the deterioration of liver disease and liver failure. After stopping cefminox sodium, the patient's fatigue gradually alleviated, the spirit and appetite gradually improved, and the coagulation function improved, which was mistakenly thought the improvement was caused by plasma transfusion, and then the general condition improved. Therefore, on August 4, when the patient suffered from peritonitis again, cefminox sodium was applied again for anti-infective treatment according to experience, and the same performance appeared as the first application of cefminox sodium. At the same time, it was found that the coagulation function was still deteriorating sharply during the second application of cefminox sodium with supportive treatment. However, after cefminox sodium was discontinued, the coagulation function was improved very quickly even without plasma transfusion. During the first and second application of cefminox sodium, the serum bilirubin levels didn’t change significantly when the coagulation function deteriorated sharply (Fig. 2), which suggested that the deterioration of coagulation function wasn’t caused by the deterioration of liver disease, but by cefminox sodium. Unfortunately, we mistakenly diagnosed that the deterioration of coagulation function was related to liver failure, so we only treated the patient with intermittent plasma transfusion. We didn't supplement vitamin K because we didn't realize the deterioration of coagulation function caused by vitamin K deficiency. In addition, our patient's liver function was classified as Child Pugh C, and vitamin K usage was forbidden for those with severe liver diseases or poor liver function. We didn’t change antimicrobials because we didn’t realize the coagulopathy side effect of cefminox sodium. Fortunately, we didn’t use cefminox sodium for a long time due to its good anti-infective effect. After stopping the drug, the patient's coagulation function quickly recovered and there were no fatal consequences happened.

Most of the coagulation factors or their intermediates are synthesized by the liver. FIB is synthesized by hepatocytes, prothrombin is synthesized by hepatocyte microsomes, and coagulation factors VII, X, VIII, IX and V may be synthesized in the liver. Patients with liver diseases, especially those with cirrhosis and liver failure, are often accompanied with coagulation dysfunction [13], and such patients are often complicated with peritonitis. In cases of further deterioration of coagulation function caused by β-lactam antibiotics represented by cefminox sodium, the Naranjo score [14] and other liver function indexes (especially serum bilirubin levels) of patients can be combined to determine whether the deterioration of coagulation function caused by liver disease deterioration or by antibiotics. If it is caused by drugs, the drug should be stopped or other kinds of antibiotics should be replaced in time.

We should not regard all cases of the coagulation function deterioration (such as PT prolonging, PTA declining, INR decreasing, etc.) as the aggravation of liver disease or liver failure in medical practice. If the patient is in the course of β-lactam antibiotics administration, his/her coagulation function deteriorates rapidly but there are no matching performances such as rapid increase of serum bilirubin levels and aggravation of general conditions, the coagulation dysfunction caused by antibiotics should be considered. Protein induced by vitamin K absence or antagonist-II (PIVKA-II) can be detected to judge whether it is VitK-dependent hypoprothrombinemia caused by antibiotics [15, 16]. If the side effect of antibiotics is confirmed or highly suspected, stopping or changing antibiotics in time can effectively avoid the occurrence of malignant events. When liver function of the patient permits, VitK injection can promote the rapid recovery of coagulation function [17]. This case provides a warning role to our doctors, especially hepatologists. We should break the mindset and broaden the thinking model in the clinical diagnosis and treatment process.

## Data Availability

Derived data are available on reasonable request.

## Abbreviations

ESBL: Extended spectrum β lactamases
PT: Prothrombin time
APTT: Activated partial thromboplastin time
PTA: Prothrombin activity
INR: International normalized ratio
TT: Bleeding time
FIB: Fibrinogen
VitK: Vitamin K
NMTT: *N*-Methylthiotetrazole
ALT: Alanine aminotransferase
AST: Aspartate aminotransferase
TBIL: Total bilirubin
DBIL: Direct bilirubin
PIVKA-II: Protein induced by vitamin K absence or antagonist-II
